# *Entamoeba moshkovskii* Infections in Children in Bangladesh

**DOI:** 10.3201/eid0905.020548

**Published:** 2003-05

**Authors:** Ibne Karin M. Ali, Mohammad Bakhtiar Hossain, Shantanu Roy, Patrick F. Ayeh-Kumi, William A. Petri, Rashidul Haque, C. Graham Clark

**Affiliations:** * London School of Hygiene and Tropical Medicine, London, England; † International Centre for Diarrheal Disease Research, Dhaka, Bangladesh; ‡ University of Ghana Medical School, Accra, Ghana; § University of Virginia School of Medicine, Charlottesville, Virginia, USA; 1 These authors contributed equally to this work.

**Keywords:** amebiasis, PCR, diagnosis, polymorphism, research

## Abstract

*Entamoeba moshkovskii* cysts are morphologically indistinguishable from those of the disease-causing species *E. histolytica* and the nonpathogenic *E. dispar*. Although sporadic cases of human infection with *E. moshkovskii* have been reported, the organism is considered primarily a free-living ameba. No simple molecular detection tool is available for diagnosing *E. moshkovskii* infections. We used polymerase chain reaction (PCR) to detect *E. moshkovskii* directly in stool. We tested 109 stool specimens from preschool children in Bangladesh by PCR; 17 were positive for *E. histolytica* (15.6%) and 39 were positive for *E. dispar* (35.8%). In addition, we found that 23 (21.1%) were positive for *E. moshkovskii* infection, and 17 (73.9%) of these also carried *E. histolytica* or *E. dispar*. The high association of *E. moshkovskii* with *E. histolytica* and *E. dispar* may have obscured its identification in previous studies. The high prevalence found in this study suggests that humans may be a true host for this ameba.

*Entamoeba moshkovskii*, considered to be primarily a free-living ameba, is indistinguishable in its cyst and trophozoite forms from *E. histolytica* (the cause of invasive amebiasis) and *E. dispar* (a common noninvasive parasite), except in cases of invasive disease when *E. histolytica* trophozoites may contain ingested red blood cells. *E. moshkovskii* has so far rarely been shown to infect humans; however, the organism appears to be ubiquitous in anoxic sediments. Although the early isolations of this species were from sewage, *E. moshkovskii* can also be found in environments ranging from clean riverine sediments to brackish coastal pools (*1*). *E. moshkovskii* is osmotolerant, can be cultured at room temperature, and is resistant to emetine, all characteristics that distinguish it from *E. histolytica* and *E. dispar* (*2*–*5*). Human isolates of *E. moshkovskii* to date have come from North America, Italy, South Africa, and Bangladesh, and they have never been associated with disease (*5*,*6*). However, few studies have actually set out to identify such infections (*7*).

The structural resemblance of the apparently innocuous *E. moshkovskii* to the disease-causing *E. histolytica* makes differentiating the two species important. In the clinical setting, for example, an *E. moshkovskii*–infected patient could be diagnosed as infected with *E. histolytica* and be treated unnecessarily with antiamebic chemotherapy. Most studies that have investigated the prevalence of *E. histolytica* and *E. dispar* have not considered the possible presence of *E. moshkovskii,* partly because of a lack of tools to detect *E. moshkovskii* other than cultivation, which is labor-intensive, not always successful, and problematic in the case of mixed infections*.* We report for the first time the application of tools to detect the species directly in stool and investigate the prevalence of *E. moshkovskii* in humans, a group of children in an *E. histolytica–*and *E. dispar*–endemic area where the first human infection with *E. moshkovskii* from Bangladesh was detected (*6*).

## Materials and Methods

### Stool Specimens

Fecal specimens included in this study were from 109 preschool children ages 2–5 years from Mirpur, an urban slum in Dhaka, Bangladesh. Based on results of polymerase chain reaction (PCR) on stool DNA samples, 39 were *E. dispar*–positive, 17 were *E. histolytica–*positive, and 1 was positive for both *E. histolytica* and *E. dispar*. Of the 52 samples negative by stool PCR, 18 were eventually found positive for *E. histolytica*, *E. dispar,* or both, either by PCR from culture DNA or by antigen detection tests performed on stool specimens, and the remaining 34 samples were negative by all methods. Only four of the samples were from children with diarrhea.

### Cell Culture and Isoenzyme Analysis

All stool samples were cultured for *Entamoeba* species in Robinson’s medium (*8*) within 6 hours of collection, and hexokinase isoenzyme analysis was performed when possible as previously described (*9*). *E. moshkovskii* strains Laredo and FIC were maintained axenically in LYI-S-2 medium (*10*) with 10% adult bovine serum. Laredo (ATCC 30042) is a human isolate, and FIC (ATCC 30041) is an environmental isolate. *E. histolytica* HM-1:IMSS clone 9 (ATCC 50528) and *E.* *dispar* SAW760 (ATCC 50484) were used as controls.

### Antigen Detection Tests for *E. histolytica* and *E. dispar*

The TECHLAB, Inc. (Blacksburg, VA) *Entamoeba* test (designed to detect but not differentiate *E. histolytica* and *E. dispar* antigen in stool specimens) and *E. histolytica* test (designed to detect specifically *E. histolytica* in stool specimens) were performed on stool specimens according to the manufacturer’s instructions (*9*).

### Preparation of DNA

Stool DNA was isolated by using a modified version of the silica-DNA binding method of Katzwinkel-Wladarsch et al. as previously described (*11*,*12*). Culture DNA was isolated by a cetyltrimethylammonium bromide (CTAB) extraction method as previously described (*13*), dissolved in 10 mM Tris-Cl (pH 8.5), and passed over a Microspin S-200 HR column (Amersham Biosciences UK Ltd, Chalfont St. Giles, England). RNA was removed by the addition of RNase A (Promega UK, Ltd, Southampton, England) to 0.05 µg mL^-1^.

### Small Subunit rRNA Gene Amplification

Based on the sequences of the small subunit rRNA genes (SSU-rDNA) of *E. histolytica* and *E. dispar*, nested sets of primers (designated E-1/E-2, Eh-1/Eh-2, and Ed-1/Ed-2) were used, as described (*11*), to detect *E. histolytica* and *E. dispar* in stool specimens (Table 1). Based on the sequence of the SSU-rDNA gene of *E. moshkovskii* Laredo (GenBank accession no. AF 149906), a nested set of primers (designated Em-1/Em-2 and nEm-1/nEm-2) was designed (unpub. data) and used to detect *E. moshkovskii* in stool DNA (Table 1). In the initial PCR (total vol. 25 µL), 1.0 µL of stool or culture DNA was used. Thermal cycler conditions included 30 cycles, each consisting of 92°C for 1 min, 55°C for 1 min, and 72°C for 1 min, followed by a final extension of 7 min at 72°C. In the nested PCR, 1.0 µL of first PCR product was used as the template DNA and the annealing temperature was raised to 62°C, leaving the other parameters of the amplification cycles unchanged. *E. moshkovskii*–specific nested SSU-rDNA gene amplification products were digested with restriction endonuclease *Xho*I for 1 h at 37°C according to the manufacturer’s instructions (Invitrogen Corp., Carlsbad, CA) to verify species identity. All PCR products were separated in 1.8% NuSieve 3:1 agarose gels (Flowgen, Lichfield, England) in 1x Tris-borate-EDTA buffer and visualized after staining with ethidium bromide (0.2 µg mL^-1^; Sigma-Aldrich Co. Ltd, Poole, England).

**Table 1:** Oligonucleotide primers used to detect *Entamoeba histolytica* and *E. dispar* in stool specimens

Primer	Primer sequence (5´ to 3´)
E-1	TTT GTA TTA GTA CAA A
E-2	GTA [A/G]TA TTG ATA TAC T
Eh-1	AAT GGC CAA TTC ATT CAA TG
Eh-2	TTT AGA AAC AAT GCT TCT CT
Ed-1	AGT GGC CAA TTT ATG TAA GT
Ed-2	TTT AGA AAC AAT GTT TCT TC
Em-1	CTC TTC ACG GGG AGT GCG
Em-2	TCG TTA GTT TCA TTA CCT
nEm-1	GAA TAA GGA TGG TAT GAC
nEm-2	AAG TGG AGT TAA CCA CCT
Arg^TCT^-1	AGC ATC AGC CTT CTA AGC TG
Arg^TCT^-2	CTT CCG ACT GAG CTA ACA AG
EmR-1	GGC GCC TTT TTT ACT TTA TGG
EmR-2	GCT AAC AAG GCC AAT CGA TAA A

### Arg^TCT^ Gene PCR Amplification

Based on the sequence of the Arg^TCT^ tRNA gene of *E. histolytica*, a set of primers were designed (Arg^TCT^-1 and Arg^TCT^-2). Thermal cycler conditions for PCR were the following: 30 cycles each, consisting of 94°C for 1 min, 55°C for 1 min 30 s, and 72°C for 2 min, followed by a final extension of 5 min at 72°C. The Arg^TCT^ amplification products from *E. moshkovskii* Laredo, *E. moshkovskii* MS15-3646 (one of the infections detected above), and *E. dispar* SAW760 were cloned into the pGEM-T Easy vector (Promega) and sequenced (MWG Biotech Ltd, Milton Keynes, England). From the sequence results, an *E. moshkovskii*–specific primer pair, EmR-1 and EmR-2, was designed to amplify the *E. moshkovskii* Arg^TCT^ gene fragment specifically (Table 1). PCR amplification was performed at an annealing temperature of 58°C as described for Arg^TCT^ gene amplification.

## Results

### Culture and Isoenzyme Analysis

All 109 stool specimens were added to Robinson’s medium for growth of *Entamoeba* species. Incubation led to growth of *E. histolytica*/*E. dispar*/*E. moshkovskii* in 33 cultures and *E. coli* in 8 cultures (no growth of *E. hartmanni* or *Endolimax nana* or was observed). Hexokinase isoenzyme analysis was possible for 10 cultures; 4 of them showed the band pattern of *Entamoeba histolytica*, 5 showed *E. dispar,* and 1 showed the band pattern of *E. dispar* with an extra band just behind the faster moving band, perhaps indicating a mixed culture with *E. moshkovskii*.

### Detection of *E. moshkovskii* by Nested PCR

**Figure 1:**
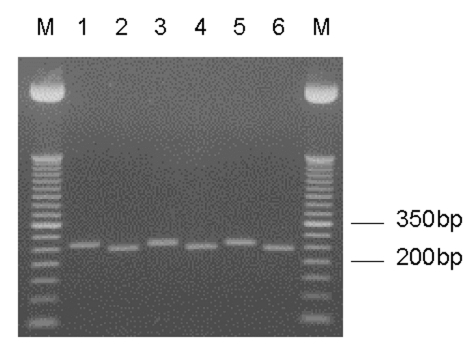
*Entamoeba moshkovskii*–specific nested SSU rDNA polymerase chain reaction (PCR) products. Odd- and even-numbered lanes represent undigested and *Xho*I-digested PCR products, respectively. Lanes 1/2, *E. moshkovskii* Laredo; lanes 3/4–5/6, DNA from stool samples. M, a 50-bp DNA ladder (Invitrogen Corp.).

The reference strain *E. moshkovskii* Laredo gave the expected band at approximately 260 bp with the *E. moshkovskii–*specific SSU-rDNA nested primers, whereas control *E. histolytica* HM-1:IMSS and *E. dispar* SAW760 DNAs were negative. Twenty-three of 109 (21%) stool DNA samples were positive by nested PCR for *E. moshkovskii* (Table 2). Of these, seven were positive for amebae by culture; one DNA sample extracted from these cultures was positive for *E. moshkovskii*. Seventeen of the 23 *E. moshkovskii*–positive samples were also positive for *E. histolytica, E. dispar,* or both, by either PCR of stool SSU rDNA (13/17) or by TECHLAB *Entamoeba* or *E. histolytica* tests (15/17) (Figure 1). One of the four children with diarrhea was positive for *E. moshkovskii* and coinfected with *E. dispar.* The cause of his diarrhea remained undetermined.

**Table 2:** Nested SSU rDNA polymerase chain reaction (PCR) (for *Entamoeba histolytica,* *E. dispar*, or both) and stool antigen-detection test results of the 17 *E. moshkovskii*–positive samples^a^

Samples	Stool antigen-detection test results:	SSU rRNA gene PCR for *E. histolytica/E. dispar*	
		Stool DNA	Culture DNA
1^b^	*E. histolytica*	*E. dispar*	Mixed
2	*E. dispar*	0	NC
3	*E. dispar*	*E. dispar*	NC
4	*E. histolytica*	0	NC
5	*E. dispar*	*E. dispar*	*E. dispar*
6^c^	*E. dispar*	Mixed	*E. dispar*
7	*E. dispar*	*E. dispar*	NC
8	*E. dispar*	*E. dispar*	NC
9	*E. dispar*	*E. dispar*	NC
10	0	*E. dispar*	NC
11	*E. dispar*	*E. dispar*	NC
12	*E. dispar*	0	NC
13^c^	*E. dispar*	*E. histolytica*	NC
14	0	0	*E. dispar*
15	*E. dispar*	0	*E. dispar*
16	0	0	*E. dispar*
17	*E. histolytica*	0	NC
18	0	0	NC
19	0	0	NC
20	0	0	NC
21	0	0	NC
22	0	0	NC
23	0	0	NC

^a^NC, no culture; 0, negative. All stool antigen tests that are positive for *E. histolytica* can also be mixed because no specific *E. dispar* antigen test exists. ^b^Patients 1 and 6 likely had mixed infections with *E. histolytica* and *E. dispar*, in which *E. histolytica* was much lower in number than *E. dispar* in the stool specimen. For patient 1, SSU rDNA PCR failed to detect *E. histolytica*, though both species grew in the culture. For patient 6, although SSU rDNA PCR could detect *E. histolytica* in stool DNA, the *E. histolytica* antigen-detection test failed to detect *E. histolytica,* and only *E. dispar* survived in the culture. ^c^The stool specimen of patient 13 was marginally negative by the *E. histolytica* antigen-detection test (optical density value was 0.13 where the cut-off value for a positive result was 0.15).

**Figure 2:**
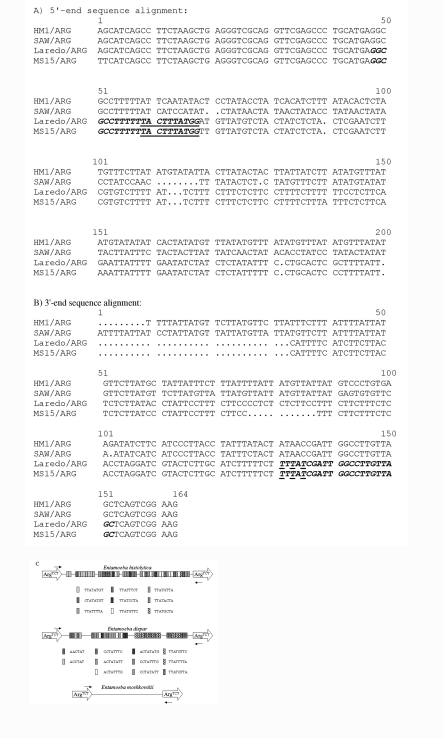
Arg^TCT^ locus. Arg^TCT^ sequences from *Entamoeba histolytica* HM-1:IMSS (GenBank accession no. AZ535059), *E. dispar* SAW760 (GenBank accession no. AF 525284), *E. moshkovskii* Laredo (GenBank accession no. AF 525285), and MS15-3646 (GenBank accession no. AF525286) were aligned at the 5´(A) and 3´ (B) ends to design *E. moshkovskii*–specific primers. The EmR primer sequences are shown in italic and bold with *E. moshkovskii*–specific positions underlined. C. Schematic representation of Arg^TCT^ loci from *E. histolytica* HM-1:IMSS, *E. dispar* SAW760, and *E. moshkovskii.* Locations of the primers used in polymerase chain reaction amplification are indicated by small arrows, the tRNA genes are indicated by large arrows, and the short tandem repeats by shaded boxes (not to scale.)

A comparison of SSU-rDNA sequences from *E. moshkovskii*, *E. histolytica*, and *E. dispar*, showed that the restriction endonuclease *Xho*I cuts exclusively in the *E. moshkovskii*–specific, 258-bp–nested PCR product to produce 236-bp and 22-bp fragments. Products from all 23 positive stool samples and the Laredo strain showed the presence of this site (Figure 2).

### Arg^TCT^ PCR and Sequence Analysis

To detect polymorphism among the *E. moshkovskii* samples, we studied a locus known to show polymorphism in *E. histolytica* and *E. dispar* (unpub. data). The Arg^TCT^ primers amplify *E. histolytica*, *E. dispar,* and *E. moshkovskii* DNA. The sizes of the PCR products from *E. histolytica* HM-1:IMSS, *E. dispar* SAW760, and *E. moshkovskii* Laredo were 586 bp, 586 bp, and 323 bp, respectively. We did not observe a band in the 250- to 350-bp region in any of the *E. histolytica* and *E. dispar* strains with these primers (data not shown). Because 17 of 23 *E. moshkovskii*–positive samples were also positive for *E. histolytica, E. dispar* (by SSU-rDNA PCR or TECHLAB enzyme-linked immunosorbent assay), or both, we ignored products in the 500- to 600-bp region (assuming that they were derived from *E. histolytica* or *E. dispar* DNA) and considered a sample positive for *E. moshkovskii* when it produced a band at approximately 300 bp. By this criterion, we found 18 of 23 samples were positive for *E. moshkovskii*, and they showed slight PCR product size variation (data not shown). The PCR products from one stool sample, *E. moshkovskii* Laredo and *E. dispar* SAW760, were cloned, sequenced, and aligned with that of *E. histolytica* HM-1:IMSS, and *E. moshkovskii*–specific primers (EmR-1 and EmR-2) were designed (Figure 2) In addition to notable PCR product size differences, analysis clearly showed that the *E. moshkovskii* sequence is completely different from those of *E. histolytica* and *E. dispar* and, unlike the *E. histolytica* and *E. dispar* sequences, it contains no short tandem repeat sequences (Figure 2C).

**Figure 3:**
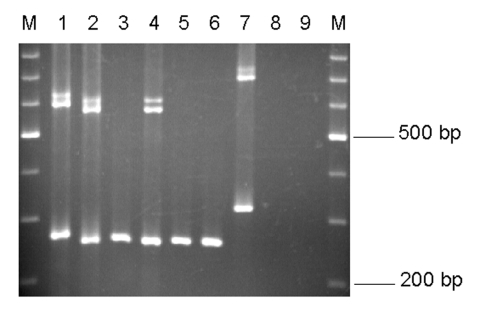
EmR polymerase chain reaction products. Lane 1, *Entamoeba moshkovskii* Laredo; lane 2, *E. moshkovskii* ICDDRB:717; lanes 3–6, *E. moshkovskii*–positive stool DNA samples; lane 7, *E. moshkovskii* FIC; lane 8, *E. histolytica* HM-1:IMSS; and lane 9, *E. dispar* SAW760. M, a 100-bp DNA ladder (Promega).

The EmR primers amplified the expected 265-bp fragment from *E. moshkovskii* Laredo DNA and did not amplify *E. histolytica* HM-1:IMSS or *E. dispar* SAW760 DNA. However, they successfully amplified 10 of a possible 23 *E. moshkovskii*–positive stool DNA samples. The most likely reason why these primers did not amplify the other 13 *E. moshkovskii* DNA samples is that they differed in sequence in the primer-binding regions. Although the PCR product size of the 10 positive samples was slightly different from that of Laredo, they were very similar in size to each other (Figure 3). The DNA of the previously reported *E. moshkovskii* ICDDRB:717, isolated from humans in the same geographic location (*6*), also gave a product of the same size (Figure 3, lane 2). The EmR primers successfully amplified DNA from environmental *E. moshkovskii* isolate FIC, but its product size was quite different from that of the human isolates of *E. moshkovskii* (Figure 3, lane 7).

## Discussion

The main objectives of this study were to develop molecular tools to identify *E. moshkovskii* and to investigate its prevalence and diversity in humans. We were successful in developing a simple diagnostic technique: a nested SSU-rDNA PCR followed by restriction endonuclease digestion. We chose to use nested PCR to detect *E.* *moshkovskii* infections because our previous experience in this area showed that nested PCR was much more efficient in amplifying stool DNA (*14*). Our attempt to produce a species-specific polymorphic marker was not completely successful. The EmR primers failed to amplify 13 of 23 *E.* *moshkovskii*–containing samples, probably because of sequence differences in primer-binding sites. However, the Arg^TCT^ primers, originally designed to amplify *E. histolytica* and *E. dispar* DNA, did amplify most of the *E.* *moshkovskii* samples, producing a product distinct in size from those of *E. histolytica* and *E. dispar*.

Our study has some limitations. The subjects were children 2–5 years of age, so we do not know whether these subjects are representive of all age groups. All previous human isolates of *E.* *moshkovskii* have belonged to ribodeme 2 (*5*). Our attempts to perform riboprinting on these infections were unsuccessful, likely because of the size of the amplification target (approximately 1.95 kb). Even if PCR had been successful, the presence of mixed infections with other eukaryotes would have prevented successful typing.

This study has several important findings. The overall *E.* *moshkovskii* prevalence (21%) suggests that this infection is common among these children. *E. dispar–*infected children were almost twice as likely to have a mixed infection with *E.* *moshkovskii* (35%) compared to those with (18%) or without *E. histolytica* (18%) infections. None of the six children with *E.* *moshkovskii* monoinfections had diarrhea or dysentery, which suggests that *E.* *moshkovskii* is a noninvasive parasite. The high prevalence of *E.* *moshkovskii* infection may have been unnoticed over the years because most such infections (74%) were mixed infections with *E. histolytica*, *E. dispar*, or both. Previous attempts to identify human *E. moshkovskii* infections (*7*) may have failed because the human intestinal flora was unsuitable for cultivation at room temperature.

The high prevalence of *E.* *moshkovskii* shown in this study population indicates that perhaps humans are a true host for this putatively free-living ameba and are not just transiently infected. This prevalence may also explain some of the microscopy- positive/antigen-negative results obtained when using the *Entamoeba* test kit (*15*). Epidemiologic studies of *E. histolytica* infection should include tools to diagnose all three of these species individually, simultaneously, and accurately, and the prevalence of *E. moshkovskii* infection in other regions of the world should be investigated.
